# *Drosophila* Accessory Gland: A Complementary In Vivo Model to Bring New Insight to Prostate Cancer

**DOI:** 10.3390/cells10092387

**Published:** 2021-09-10

**Authors:** Amandine Rambur, Marine Vialat, Claude Beaudoin, Corinne Lours-Calet, Jean-Marc Lobaccaro, Silvère Baron, Laurent Morel, Cyrille de Joussineau

**Affiliations:** 1 Rosalind and Morris Goodman Cancer Research Centre, McGill University, 1160 Pine Avenue West, Montréal, QC H3A 1A3, Canada; 2 Institut de Génétique, Reproduction et Développement, Université Clermont Auvergne, CNRS UMR6293, INSERM U1103, 28 Place Henri Dunant, BP38, 63001 Clermont-Ferrand, France; marine.vialat@uca.fr (M.V.); claude.beaudoin@uca.fr (C.B.); corinne.lours@uca.fr (C.L.-C.); j-marc.lobaccaro@uca.fr (J.-M.L.); silvere.baron@uca.fr (S.B.); laurent.morel@uca.fr (L.M.)

**Keywords:** prostate cancer, *Drosophila*, early tumorigenesis, late tumorigenesis, in vivo model

## Abstract

Prostate cancer is the most common cancer in aging men. Despite recent progress, there are still few effective treatments to cure its aggressive and metastatic stages. A better understanding of the molecular mechanisms driving disease initiation and progression appears essential to support the development of more efficient therapies and improve patient care. To do so, multiple research models, such as cell culture and mouse models, have been developed over the years and have improved our comprehension of the biology of the disease. Recently, a new model has been added with the use of the *Drosophila* accessory gland. With a high level of conservation of major signaling pathways implicated in human disease, this functional equivalent of the prostate represents a powerful, inexpensive, and rapid in vivo model to study epithelial carcinogenesis. The purpose of this review is to quickly overview the existing prostate cancer models, including their strengths and limitations. In particular, we discuss how the *Drosophila* accessory gland can be integrated as a convenient complementary model by bringing new understanding in the mechanisms driving prostate epithelial tumorigenesis, from initiation to metastatic formation.

## 1. Introduction

Prostate cancer (PC) is the most prevalent cancer affecting older men in developed countries. It is characterized by the uncontrolled, malignant growth of cells in the gland, which ultimately cause symptoms like pain or trouble with urination and ejaculation [1]. Similar to other carcinomas, the first steps of the disease are asymptomatic, rendering early detection difficult without a preventive medical intervention. Therefore, most prostate cancers are detected in late stages, years after disease initiation, and when the benchmark treatment is hormonotherapy. Prostate cancer cells depend on androgens to maintain growth and proliferation, and due to this dependency, androgen deprivation therapy has been shown to be highly effective. However, after an initial response, treatment resistance occurs, and patients develop castration-resistant prostate cancer (CRPC) for which aggressive treatment can slow but not cure the disease. Hence, CRPC ultimately leads to death. For these reasons, improving the prevention and diagnosis of prostate cancer is essential for better patient care. It is now well known that prostate cancer is a heterogeneous cancer, with particularly different evolutions and treatments needed depending on the tumor aggressiveness. Some tumors are indeed very aggressive and will progress quickly, while others are indolent for years or decades, and ideally just require monitoring without curative treatment. Because of this heterogeneity, it is difficult to determine which genetic abnormalities cause prostate cancer initiation, progression, and ultimately treatment resistance. 

In more than 95% of cases, prostate cancer is adenocarcinoma, i.e., of epithelial origin, whereas <2% of cases come from neuroendocrine cells [2]. The formation and evolution of adenocarcinomas follows a classic process that has been summarized in three stages: (1) initiation, in which genetic mutations are thought to be the main driver; (2) promotion, during which an accumulation of more genetic events take place, and where uncontrolled proliferation begins; and (3) progression, which in fact includes different steps of tumor evolution, the most significant being early invasion leading to formation of localized prostate cancer, and then, late invasion. Late invasion corresponds to formation of metastatic prostate cancer, when cells have acquired independence from their environment and are able to form secondary tumors mainly in lymph nodes, bones, brain, lungs, and liver. The evolution of adenocarcinoma through these different phases is the consequence of a succession of genetic alterations (loss of chromosomal fragments, gene fusion, or mutations), and activation of signaling pathways, particularly those involving growth factors, as well as a modification of the microenvironment. Several alterations are associated with prostate cancer. Among them, loss of expression of the tumor suppressor gene NKX3.1 is found in 60–80% of prostate tumors [3,4], and is caused by loss of its heterozygosity or inhibitory epigenetic events, including methylation of its promoter [5]. The most common gene fusion is TMPRSS2-ERG, combining the genes encoding ERG (ETS-related gene) and the serine protease TMPRSS2, found in about 50% of localized prostate cancers [6,7]. Finally, mutation or deletion of genes, such as PTEN, are found in 50-80% of prostate cancers, and result in the overactivation of the PI3K/AKT/mTOR signaling pathway [8,9,10]. Even though the understanding of the main mechanisms involved in prostate cancer is progressing, it is still necessary to determine and study the key alterations responsible for the initiation and progression of prostate cancer through its different stages. Genomic studies have revealed that primary prostate cancer cells display more than 2000 genetic alterations, whereas around 9000 alterations are detected for CRPC [11]. Furthermore, in some cases, cancer evolution can be correlated to different genomic profiles [12], showing both the actual diversity of these 2000–9000 alterations depending on the considered patient and the necessity to better understand their respective role in order to improve treatments according to a patient’s key alterations. Of course, these studies require pertinent and complementary models to overlap all aspects of prostate cancer biology. Here, we will present the principal cell lines and mouse models that have been developed as specific prostate cancer models, and their relevance for the study of the different stages of the disease. As part of a special issue, we will further present how *Drosophila* models are increasingly used to better understand fundamental biological processes implicated in tumorigenesis and how they could allow the study of key stages of carcinogenesis that are still poorly understood. Finally, we will discuss the necessity of utilizing multiple and diverse models in order to study the many tumor evolutions that are included in the generic “prostate cancer” term.

## 2. Cell Lines and Mouse Models of Prostate Cancer Disease

### 2.1. Two-Dimensional and 3-D Cell Culture for Deciphering Molecular Mechanisms

Since the first cell line derivation in 1975, more than 200 cell lines have been developed and used for PCa studies [13,14]. Each cell line displays a specific molecular signature, and it is important to take it into account in order to choose the most relevant model to answer the chosen biological question. Among the most important molecular markers to consider is the presence or absence of the androgen receptor, which determines if the cells are androgen sensitive, and representative of an earlier stage of tumorigenesis, or androgen insensitive, and representative of late-stage CRPC. Another factor is the expression of PSA (prostate-specific antigen), a protein produced by prostate epithelial cells and used to diagnose prostate cancer. Finally, it is important to consider the presence or absence of genetic alterations characteristic of PCa, such as those previously mentioned (deletion of NKX3.1, loss of PTEN, etc.). Prostate cell lines can be classified into three main groups: untransformed, androgen-sensitive, and androgen-insensitive/castration-resistant cells.

Only a few prostate cell lines are immortalized but non-transformed, and therefore non-tumoral. The P69SV40-T (P69) cell line is derived from the normal prostate gland of a 63-year-old man. Interestingly, this cell line is immortalized and responsive to IGF1, yet it is not transformed [15]. The RWPE-1 cell line is derived from the peripheral zone of a normal adult human prostate and expresses the androgen receptor as well as PSA [16]. In contrast, the BPH-1 and pRNS-1-1 lines, derived from primary prostate tissue and radical prostatectomy, respectively, express neither the androgen receptor nor PSA [17,18]. These cell lines allow the study of normal growth and development of the prostate [19]. They also allow the study of non-tumorigenic prostatic pathologies, such as benign prostatic hyperplasia (BPH), the most frequent pathology affecting the prostate, which corresponds to a non-malignant excessive growth of the organ [20].

Prostate cancer cell lines are either derived from primary or metastatic tumors, or clonally derived from the previously established cell lines. However, because prostate cancer is characterized by slow growth, it is difficult to obtain cell lines from a primary tumor, and most of the lines are derived from metastases. Furthermore, such cells lines, for example 1013L or E006AA, are less aggressive than those derived from metastases and are rarely used for research purposes [21,22]. Prostate cancer cell lines derived from metastases come from different sites and represent a large panel of the heterogeneity at this stage of disease, including different mutations, gene expression, shape, and metastatic potential. Some cell lines are still sensitive to androgens and thus represent an earlier stage of prostate cancer development. The most representative cells for this stage are LNCaP cells, isolated in 1980 from a lymph node metastasis, and expressing a mutated form of androgen receptor (T877A) [23]. Other androgen-dependent cell lines include LAPC-4, LAPC-9, or LuCaP 23.1, and are derived from lymph node or bone metastases [24,25,26]. All these cell lines have specific molecular characteristics that must be taken into consideration when used: LNCaP and LAPC-9 are deficient for PTEN, and display a constitutively active PI3K/Akt pathway, while LAPC-4 has a mutation in the P53 tumor suppressor gene. Cell lines that are not sensitive to androgens are representative of a later stage of prostate tumorigenesis, known as CRPC, which generally appears after hormone therapy. This is the case for the PC3 cell line. Isolated from vertebrae metastasis in 1979, this cell line is capable of forming a tumors that metastasize to the lungs, liver, and kidney when injected into mice [27]. The DU145 cell line, derived from a brain metastasis in a 69-year-old white man in 1975, is also androgen independent and tumorigenic when injected in mice [28]. DU145 cells grow well in vitro and can lead to metastasis in mice after injections, depending on the microenvironment [29,30]. Along with LNCaP and PC3, the DU145 constitutes the most utilized PCa cell culture lines. The PC3 and DU145 cell lines are not sensitive to androgens because they no longer express the androgen receptor. However, during the progression of prostate cancer, the acquisition of this androgen independence can occur via loss of androgen receptor expression or via other mechanisms that make the receptor active independently of androgens, i.e., gain-of-function mutations in the receptor [31,32]. In order to study the impact of such mechanisms on prostate cancer evolution, new cell lines can be obtained by androgen deprivation, transfection, co-culture, xenograft, or chemical mutagenesis of the already available cell lines. The LNCaP cells, for example, are androgen dependent and poorly tumorigenic in mice. However, castration-resistant lines expressing the androgen receptor, the C4-2 and C4-2B lines, were derived from them. This was done by xenografts of LNCaP cells combined with osteosarcoma cells in mice, then castration of these mice, and then collection of vertebral and bone metastases, respectively [33,34,35,36]. Utilizing this method for generating new cell lines is essential to study the different aspects of resistance to hormonotherapy, while also providing cell lines with higher genetic abnormalities, tumorigenic capacities, and metastatic potential.

The use of cell lines allows for the study of molecular interactions and alterations of cancer cells, and ultimately provides functional and mechanistic insight. They are well characterized and relatively easy to obtain. They can be manipulated to allow inhibition or overexpression of genes of interest, as well as the expression of fluorescent markers. Furthermore, their proliferation, migration, invasion, and cell adhesion capacities can be analyzed. Thus, they facilitate the study of alterations found in cancer cells, from establishing which molecular mechanisms are altered when these alterations are present, to how these alterations modify the behavior of the cells. In this way, they allow for the in vitro study of certain biological processes that are associated with the passage from an early tumorigenic stage to a more advanced stage in vivo. Commonly used tests are wound healing assays for migration evaluation, spreading assays to assess cell adhesion, and Transwell assays for testing migration and invasion [37].

The main disadvantage of cell lines is their inability to reproduce the pathology found in whole organisms. First, because most cell lines are derived from metastases, they present many genetic alterations, which limits their use for the study of early prostate cancer mechanisms. Moreover, the fact that these cells were cultured for years may have induced cell derivation and altered their initial representative nature of prostate cancer. Finally, complex cellular interactions in the tumor microenvironment are essential for cancer initiation and progression, and traditional bi-dimensional cell culture (2-D), where only one type of cell grows as a monolayer, does not model this rich environment [38,39]. 

The 2000s witnessed the emergence of three-dimensional (3-D) cell culture models. These models can be closer to a native tumor, with better conservation of its heterogeneity and architecture, and with partial conservation of the tumor microenvironment. Two major 3-D tumor models have been developed: spheroids and organoids [40]. While spheroids are composed of clusters of broad-ranging cells growing as free-floating structures, organoids are more complex and composed of organ-specific cells, intended to assemble in a scaffolding extracellular environment to form microscopic versions of parent organs. This multicellular combination is required for accurate reproduction of the (tumor) microenvironment and allows the conservation of many of the histological features found within the original tissues. Organoids have been obtained for a large number of organs like the colon [41,42], pancreas [43], breast [44], and lung [45]. The first organoid model of prostate cancer was made in 2014 from a patient biopsy [46]. In addition to growth in a more relevant environment, this model allows, when propagated from tumor cells, important molecular signatures of prostate cancer to be retained, such as TMPRSSE-ERG fusion or alterations in the p53 and Rb pathways. Other 3-D models of prostate cancer have since been developed from biopsies of lesions [47]. The main advantages of these structures include the conservation of cell morphology, cell–cell/cell–matrix interactions, and that they are generated from patients, allowing conservation of prostate tumor alterations that are specific to individuals. These models can be used for fast therapeutic screens without in vivo models, opening the door for precision medicine where individual patient characteristics are used to improve prevention methods, diagnosis, and personalized treatment. However, the generation of organoids is still in development, and not used as a routine procedure in most labs. Furthermore, it remains an ex vivo procedure, limiting its use for metastatic dissemination, for example. Moreover, these models mostly use aggressive cells representative of advanced-stage prostate cancer. Even though they represent a pertinent model for preclinical and late-stage studies, they are not yet effective for early stages of prostate cancer.

### 2.2. Mouse Models of Prostate Cancer 

In vivo prostate cancer models have been used for decades. They allow global studies with conservation of the interactions occurring between the prostate neoplastic cells, the stroma, and the tumoral microenvironment, including systemic (e.g., hormones, immune system) and local influence (e.g., growth factors, non-epithelial cells). These models allow direct study of genes or group of genes in the tumorigenic process and can be used for both basic and translational research. The first in vivo models used for prostate cancer were rat and dog because they developed spontaneous prostate cancer. However, the frequency for tumor development is completely random and therefore not optimal for experiments. That is why numerous procedures have been developed to produce tumors, mainly in mice, and through two major ways: introduction of genetic alterations in the prostate epithelium, and xenograft models. 

#### 2.2.1. Xenograft Mouse Models

Xenograft models consist of transplantation of prostate cancer cell lines in a mouse using three different modalities: subcutaneous, orthotopic, or in the SubRenal capsule (SRC). Subcutaneous models were developed in the 1970s and have been widely used for cancer studies. Depending on the injected cell line, the tumor can take several weeks to several months to grow. The tumor develops under the skin, rendering it accessible and easy to monitor its growth without invasive manipulation. However, the subcutaneous microenvironment has low vascularization and presents a low percentage of engraftment success. Moreover, as the subcutaneous microenvironment is very different from the prostatic microenvironment, these models are more likely closer to 3-D cell models than prostate tumor models [48]. SubRenal Capsule (SRC) transplantation has been introduced more recently to increase the efficiency of grafting in mice [49,50]. It allows for a good percentage of efficient transplantation due to the large amount of vascularization within the tissue. Notably, SRC transplantation can be done with benign and malignant tissue, while subcutaneous xenograft can only be established with high-grade tumor cells. Moreover, cells are engrafted in a definite organ where the capsule represents a real frontier. Metastatic potential can be evaluated by the ability for the xenograft cells to cross the capsule and form tumors in other distant organs. The main weakness of this transplantation procedure, like for subcutaneous transplantation, is the microenvironment where the cells are transplanted, which is very different from the prostate microenvironment. Finally, the orthotopic model consists of the introduction of prostate cancer cells directly into the mouse prostate. Unlike previous transplantation models, tumor growth occurs in the prostate microenvironment and allows for the conservation of the interactions between implanted tissue and the prostate tumoral microenvironment [51]. Orthotopic models have existed since the 1990s and can generate metastases, allowing their use for both tumorigenic and metastatic processes [52]. Furthermore, it is possible to mimic and study the acquisition of castration resistance observed in androgen-deprived patients by injection of androgen-dependent cells followed by mouse castration. With many cell lines available today, there are many possibilities to study specific prostate cancer stages. 

Overall, xenograft models appear efficient for rapid analyses of both early and late stages of prostate cancer progression, and in some cases including metastatic studies. Moreover, tumor development occurs in a whole organism with physiological processes conserved. However, we now know the major importance of the immune system in cancer development [53,54]. Additionally, as most of the xenografted cells are of human origin, immunodeficient mice are used as a recipient to avoid cell elimination by the mouse immunity system. So, the tumor microenvironment is modified, the immunobiology of prostate cancer cannot be determined with these models, and this immunodeficiency definitely impacts tumor development [55,56]. Further, when cell lines with one combination of genetic abnormalities are injected to obtain these models, the characteristic heterogeneity of prostate tumors is not preserved. More recently, the emergence of PDX models (patient-derived xenograft models), corresponding to patient tissue injection into mice, resolved this problem. The PDX models of prostate cancer are now used for anticancer drug screening [57,58] and could allow, despite the immunodeficiency of the mice, an adaptation of the experiment for a selected patient. This model facilitates the possibility of personalized medicine, with optimization of treatment occurring in the mouse model before it is used in the patient. Still, in any case, cells that are injected into mice are already transformed. As primary tumor cells already display more than 2000 genetic alterations, and CRPC cells around 10,000, it is difficult to study the mechanisms of early tumorigenesis in this context [11].

#### 2.2.2. Genetic Models in Mice

Since the 1990s, numerous genetically engineered mouse models (GEMs) have been developed to introduce specific genetic alterations in specific tissues, such as the prostate epithelium. One complication of this kind of model concerns the organization of the mouse prostate compared to the human prostate. The human prostate is composed of three zones surrounding the urethra: the central, transition, and peripherical zones [59]. The mouse prostate is organized with different lobes, by pairs, morphologically different from the human prostate zone, and also has different levels of sensibility to androgens (reflecting the androgen receptor level of expression) [60,61]. These differences in androgen sensibility can have an impact on genetic mouse models because the most specific promoter used to induce tumorigenesis in these models is based on the probasin gene, which displays prostate-specific expression and is regulated by androgen receptor signaling [62]. Moreover, it is still not completely clear which lobe is the most representative of the human prostate. The stroma surrounding the prostate also appears differently: it is very thin in mice compared to the dense fibromuscular stroma of humans [63]. Nonetheless, GEM models allow tumor growth in an intact prostate microenvironment with the presence of a normal immune system, and the effect of gene manipulations and drug treatment can be well controlled and observed temporally. As for cellular models, there are many mice designed for prostate cancer studies, because a single mouse model is not enough to overlap all aspects of prostate tumorigenesis. 

Prostate tumorigenesis is supported by mutations or alterations of tumor suppressor genes and oncogenes. Two major pathways upregulated and driving prostate carcinogenesis are p38/MAPK and PI3K/Akt signaling pathways, which are altered in more than 40% of prostate adenocarcinomas and almost all metastases [8]. It is therefore not surprising that among the most used GEM models are those displaying an inactivation of phosphatase and tensin (PTEN), an inhibitor of the PI3K/Akt pathway, or an overexpression of Ras, inducing overactivation of the p38/MAPK pathway. To generate genetic models, tools like the Cre/Loxp system are used, to have a spatiotemporal control of the genetic modification. The recombinase Cre, a nuclease from a bacteriophage, can recognize conserved sequences, referring to the Loxp sites. It can then excise all genetic information encoded between two of these sites [64]. For example, one largely used model is the PB-Cre4XPtenloxp/loxp, where CRE expression is dependent on the specific probasin prostate-specific promoter, coupled to two androgen receptor response elements (ARR2PB-Cre4). Consequently, PTEN is inactivated specifically in the prostate epithelium, inducing constitutive activation of the PI3K/AKT pathway, and ultimately prostate carcinogenesis. These tumors are similar to what is observed in humans with PIN lesions, including invasive adenocarcinoma and in some case metastases in the lymph nodes and lung [65,66]. This model can then be used to do bi/tri-genic models with, for example, a combination with deletion of known tumor suppressors, such as Nkx3.1 or p27kip1 [67,68], or overexpression of oncogenes, such as k-Ras [69] or Myc [70], to obtain a more aggressive phenotype and explore the role of the same alterations that are found in patients. Other models are developed by expression of oncogenes like the SV40 large T antigen under androgen regulation in prostate epithelium, corresponding to the TRAMP model [71]. This model is widely used because it presents development close to what is observed in humans: from PIN lesion formation to an aggressive tumor with the appearance of metastasis observed at the lymphatic level. However, in many cases, it has been observed that mice instead develop neuroendocrine prostate cancer, limiting its use for prostate adenocarcinoma studies.

The main advantage of using genetic mouse models is the preservation of an intact microenvironment and immune system. Moreover, depending on the model, the histopathological features observed in human pathology are preserved. However, depending on genetic alterations, the growth of tumors can take several weeks or months, which can make their use costly and time-consuming. Contrary to cell lines, animal model constraints limit the number of genetic alterations that can be simultaneously tested. Furthermore, gene redundancy in mammals can complicate the studies about signaling pathway interactions. There are also ethical concerns, as shown by the growing importance of the 3Rs (replacement, reduction, refinement), which push for a decrease in the use of animal models. Finally, the low rate of success of clinical trials emerging from mouse and cellular models emphasizes the necessity to still improve cancer therapeutics, for which it is necessary to develop additional and novel approaches to complement the current ones.

## 3. *Drosophila*, a Model for Human Pathologies and Prostate Cancer

The fruit fly, *Drosophila melanogaster*, is a reference model for genetic and developmental studies. *Drosophila* has several advantages compared to other models: a short life cycle (10 days at 25 °C), a large number of offspring per generation, a well-described anatomy, and vast amounts of genetic tools available. A large number of new strains can be generated rapidly for a variety of assays. Moreover, there are few redundancies compared to mammalian genomes, making the loss-of-function studies easier in this model. The fundamental biological mechanisms and signaling pathways are conserved between *Drosophila* and mammals, and 70% of genes that were found mutated, deleted, or amplified in human pathologies possess an ortholog in *Drosophila* [72,73]. The physiological function of these genes has been under investigation in *Drosophila* for the generation of human pathological models [73], and many functional studies have been done in this model to study complex signaling pathways that are relevant in pathologies, and particularly in cancer research [74]. In fact, many intracellular signaling pathways able to drive tumorigenesis and tumor microenvironment implication and interactions were first identified and characterized in *Drosophila* [75,76]. *Drosophila* was one of the first experimental models to show a lethal recessive mutation, lethal 7, which induces transplantable malignant tumor leading to lethality [77,78]. As in mammals, tumorigenesis in *Drosophila* implies cell homeostasis deregulation. The loss of function of tumor suppressor genes, such as Scribble, Disc large, or Merlin, increase cell proliferation [79,80,81]. Inhibition of the Hippo signaling pathway induces increases the expression of Cyclin E, which enhances the cell cycle, and DIAP1, an inhibitor of apoptosis [82]. It is also possible to modulate the proliferation/apoptosis balance, and consequently, to study the molecular mechanisms implicated during carcinogenesis. Regulatory mechanisms that are essential to maintain genome integrity are also well conserved in *Drosophila*. It is the case, for example, for the P53 protein, a mediator of one prominent pathway of cell survival, whose loss induces defective cell apoptosis [83]. Considering that interactions between regulatory processes and signaling pathways are at least partially conserved in *Drosophila*, it is therefore a relevant and powerful model for the study of human pathologies and cancer. It can be used differentially to study prostate cancer: to investigate conserved tumorigenesis mechanisms, for the discovery of new regulator/signaling pathways, for pharmacological screening, and for epithelial carcinogenesis modeling, including early prostate carcinogenesis.

### 3.1. Drosophila Genetic Tools and Their Use in Tumorigenesis Studies

As described by Hanahan and Weinberg in 2000 and 2011, even if each tumor displays specific features, depending on cell origin, organ, or even genetic mutations, several hallmarks of cancer are common and can be investigated independently of cancer specificity (e.g., sustained proliferation and evasion of apoptosis) [84,85]. Because fundamental processes are well conserved, *Drosophila melanogaster* can exhibit some classic hallmarks of cancer, and that is why this model is relevant for cancer investigation. In particular, three genetic tools make *Drosophila* powerful to dissect the role of signaling pathways with spatial and temporal precision: the combination of the UAS/Gal4/Gal80 binary expression system [86,87], the FLP-FRT recombinase system [88,89], and the availability of RNAi transgenic animals. UASs (upstream activation sequences) are nucleic sequences targeted by the yeast transcription activator gene gal4. Gal4 can be expressed time and tissue specifically using native *Drosophila* gene promoters, and so induces in the same pattern the expression of every sequence placed downstream of a UAS, which can encode for a fluorescent protein, an oncogene, or an RNAi, for example. Gal80 is a Gal4 antagonist that even exists in a thermosensitive version. Its co-expression permits further limits on Gal4-induced expression at the desired development time by shifting the culture temperature to 29 °C [87]. The Flp-FRT recombinase system is similar to the Cre/Loxp system used in mice: the flippase (Flp) recombines flippase recognition target sequences (FRT) and induces either chromosomal recombination or excision of the sequence, which was flanked by the FRTs. Moreover, in *Drosophila*, the flippase expression can be dependent on a heat shock promoter, hsp70, allowing for temporal control of the genetic recombination when *Drosophila* is placed at 37 °C. In this case, the length of the heat shock will determine the quantity of flippase produced, as well as the percentage of cells that will actually have enough flippase to recombine. This gives a variable level of mosaicism in the tissue, representing a unique opportunity to have within the same tissue a vast majority of normal cells alongside a few modified cells, which allows for an accurate portrayal of the microenvironment of tumor cells at the beginning of the tumorigenic process. Mosaic analysis with a repressible marker, the MARCM system [90], is a typical example of the use of this Flp/FRT systems. Amongst others, it allows the generation of spots of cells mutated for tumor suppressor genes in a heterozygous background, mimicking loss of heterozygosity, a fundamental process in tumor progression. Oncogenic activating mutations or specific gene overexpression will also induce tissue overgrowth, invasive, and metastatic behavior [91,92].

### 3.2. Drosophila, the Origin of Signaling Pathways and Gene Discoveries Relevant for Prostate Cancer

Historically, many signaling pathways, regulators, and new genes have been firstly discovered in *Drosophila* and then implicated in mammalian cell biology, including the Hedgehog, Notch, Wnt, Hippo, and Dpp signaling pathways [93,94,95]. The link between Notch deletion and developmental defects was made firstly in *Drosophila*, and we now know the importance of Notch signaling in carcinogenesis [96,97]. Numerous studies have since allowed for the discovery of new Notch interactors involved in tumorigenesis. This is the case of a study from 2014, which focuses on PTOV1, an adaptor protein able to modulate proliferation and the cell cycle, which is overexpressed during prostate cancer [98]. After showing in human prostate cancer cells that PTOV1 expression correlates with Notch targets’ repression, the authors used *Drosophila* to study the interactions between PTOV1 and Notch. Indeed, in *Drosophila,* Notch-null mutants are associated with notched wings while a Notch gain-of-function mutation is associated with a defect in the development of a wing vein [99,100]. These two phenotypes are easy to observe and allow a rapid functional analysis of Notch signaling. The authors were able to show that PTOV1 acts as a negative regulator of Notch signaling, as its expression in *Drosophila* induces the formation of notched wings and can reverse the development defects induced by a Notch gain-of-function mutation. These results were then supported by mouse experiments and analysis of human prostate tissue. This article is a great example of how complementary models (*Drosophila*, prostate cancer cells, spheroid, mouse, and human prostate samples) can be used, each giving different information, to demonstrate a new regulatory function of a protein on the Notch pathway in vitro and in vivo, and to prove its relevance to prostate cancer progression. Another example concerns the Hippo signaling pathway, which has also emerged from studies on *Drosophila* and is well known as an actor of prostate cancer tumorigenesis [101]. A major target gene of this pathway is MYC, which is overexpressed during prostate carcinogenesis [102,103]. The first tumor suppressor gene identified in *Drosophila* is lgl (lethal giant larvae), which is implicated in epithelial cell polarity, and loss of expression of which is responsible for abnormal growth of the larval brain and imaginal discs. Furthermore, when it is associated with another mutation of the same polarity complex, such as scribble, mutated tissues can induce secondary tumors [104]. A link between lgl and the Hippo signaling pathway has been made in *Drosophila* [105,106]. Moreover, homologs have been found for lgl in mammals: HUGL-1 and HUGL-2 [107], whose roles have now been extensively investigated. 

In addition to signaling pathways, some genes have been firstly discovered in *Drosophila*. It is the case for tribbles, a gene that seems to block mitotic progression during fly development, and particularly during the gastrulation stage [108]. Mitotic mechanisms and regulation are essential during carcinogenesis. This is why orthologs of this gene have been searched in other species like dog, rat, and human, followed by functional studies. SKIP3, the human ortholog of tribbles, is expressed in human tumors and in the PC3 cell line, and is regulated by hypoxia, giving it an important function during carcinogenesis [108]. Another article investigated the link between Perlecan, a basement membrane component, and the Hedgehog signaling pathway, which was first demonstrated in *Drosophila*. They show that HSPG2 (perlecan) is a new component of the SHH pathway in prostate tumorigenesis that works independently of the androgen signaling pathway [109]. This gives new perspectives for drug targeting by blocking SHH effects during prostate carcinogenesis.

Another example concerns the use of *Drosophila* cells, S2 cells, one of the most commonly used *Drosophila melanogaster* cell lines, derived from a primary culture of late-stage embryos. They have been used to do a genome-wide RNA interference screen to identify new regulators of the androgen receptor [110]. In this study, S2 *Drosophila* cells were transfected with the human androgen receptor with luciferase as an activity reporter. Then, a genome-wide RNAi screen was performed and combined with R1881 treatment to induce AR activation. The impact of RNAi on androgen receptor activity was then monitored using luciferase to identify inhibitors or enhancers of AR signaling. The existence of RNA interference libraries makes this kind of analysis quick and easy with *Drosophila* tools, before considering other analyses in human cells or in vivo models, which will be more expensive and time consuming. The discoveries done with this kind of screen allowed the identification of new regulators that are potential new targets in prostate cancer, for drug therapies.

In addition to the intrinsic characteristics of tumors, it is known that the tumor microenvironment is also essential for its progression. Interestingly, the use of imaginal discs for the study of tumorigenic phenomena shows recruitment of immune cells, a major component of the microenvironment during carcinogenesis [111,112]. The partial conservation of the immune system in *Drosophila* and the evidence of immune cell recruitment in *Drosophila*-induced tumors highlights the relevance and the numerous possibilities for studies in this model.

So, although *Drosophila* presents major differences with mammals, the conservation of many genes and signaling pathways makes it possible to translate research done in *Drosophila* to mammals. In addition, because *Drosophila* has fewer redundancies than more complex models, studies can be done easily and more quickly. Thus, *Drosophila* can be the source of studies in mammals that will advance the understanding of tumorigenic mechanisms and provide new potential therapeutic targets.

### 3.3. Drosophila Tissues Used as Models for Studying Fundamental Tumorigenic Processes

Some specific cellular processes in *Drosophila* can also be used to study mechanisms related to cancer development. This is the case of the important use of imaginal discs to model general epithelial tumorigenesis or of the tracheal network to study neo-angiogenesis.

#### 3.3.1. The Imaginal Discs

Imaginal discs are embryonic structures composed of epithelial cells able to generate adult organs, such as eyes, legs, wings, mouthparts, antenna, halteres, and the genitalia system. While adult cells are quiescent, imaginal disc cells have intact proliferative capabilities, and that is why they are widely used for carcinogenesis studies. They are determined to become specific structures, but under conditions of damage or gene mis-expression, discs can switch fate, a phenomenon called transdetermination [113]. This is due to a change in cell fate without reversion to an embryonic stage (dedifferentiation) [114]. Use of imaginal discs is interesting in cancer studies, which found that epithelial cells gain new capacities to become invasive and metastatic without complete dedifferentiation. The two main imaginal discs used for tumor growth and invasion studies are the wing and the eye discs because their modification in the larval stage induces visible phenotypes [115,116]. This is the case for the modification of Notch signaling, which was discussed earlier, and which induces notched wings or defects in the development of the wing vein. Another example related to prostate cancer is a recent study where the authors showed in mice that CDPC1 (CUB domain-containing protein 1), a transmembrane protein that is a substrate for SRC family kinase, can drive prostate cancer progression via activation of the MAPK signaling pathway [117]. In *Drosophila*, increased EGFR/Ras/MAPK signaling in wing imaginal discs induces the formation of bristles located on the dorsal part of the thorax, a tumor-like phenotype. Thus, the authors used this feature to show that CDCP1 overexpression initiates tumorigenesis in vivo. With complementary models, the authors demonstrated that CDCP1 is a powerful driver of prostate cancer progression, opening new potential therapeutic strategies. The possibility to follow rapidly identifiable phenotypes allows the use of *Drosophila* to perform screens for a chemical molecule or even new potential regulators of specific signaling pathways [118,119]. It was illustrated in a study where *Drosophila* was used as the in vivo tumorigenesis model to confirm the effects of radiosensitizing compounds that were screened initially in vitro in DU145 cells, with exploitation of the eye phenotype to evaluate the drug toxicity and effect on tumorigenesis [120]. Genotoxicity can also be done during *Drosophila* wing disc development using the fast SMART (somatic mutation and recombination test), as it was used to evaluate potential risks induced by molecules used in the treatment of benign prostate hyperplasia [121]. 

The advantage of using imaginal discs as models is that they are an easy-to-manipulate epithelial tissue. Inhibition or expression of specific genes in these discs can be done thanks to already known drivers, and phenotypic observation induced by the manipulation is directly visible by microscopy, due to the small size of the tissue, and ultimately avoids classical histological procedures that denature the tissue structure. However, this size aspect makes it difficult to perform molecular analysis, such as protein analysis, which requires a larger amount of tissue. Moreover, the major inconvenience of using imaginal discs is that they are developing tissues, with their specific activations of signaling pathways and gene expression that are inducing strong proliferation, cell migration, and cell differentiation, all processes that are tightly controlled in adult tissues and strongly targeted by the tumorigenic process. Therefore, it is preferable to use other models to validate the observations made in imaginal discs when studying tumorigenesis.

#### 3.3.2. The Tracheal System

Tumor cell growth depends on nutrient and oxygen availability [122]. In *Drosophila*, nutrient transport relies on hemolymph, a circulatory liquid, similar to blood and invertebrate interstitial liquid. Hemolymph propulsion occurs in the entire organism through the heart. The circulatory system of *Drosophila* is open and allows direct exchange of gases and nutrients/cell byproducts between hemolymph and internal organs [123]. For oxygen, *Drosophila* has an additional, sophisticated system of interconnected tubules: the tracheal system, comparable to the circulatory system of mammals [124]. It is a sensor system of oxygen level and metabolic activity and allows adaptation when environmental changes occur. During development, growth and connection of this tracheal system is supported by Fgf expression (breathless, btl), depending on the detection of hypoxia by HIF1 homolog Sima [125,126]. Neo-tracheogenesis, which can be considered as an equivalent of neo-angiogenesis, also occurs in *Drosophila* models of tumorigenesis, reportedly when oxygen levels are lowered by high tumor cell metabolism [127,128]. 

Tube formation is a universal process conserved in multicellular organisms. A large number of adult mammal organs are tubular, for example, the lung, the digestive system, or even the secretory glands, such as the pancreas or the prostate. The tubulogenesis of the tracheal system in *Drosophila* can be used to study the formation and functionality of other tubular organs. For example, cancers, such as lung cancer, have been modeled by the expression of Ras^V12^ and downregulation of PTEN to induce Ras/MAPK and PI3K/AKT signaling in the tracheal network [129,130]. This model has also been used for a drug screen to identify chemical compounds able to reduce cell proliferation and to improve tracheal physiological functions. 

Mechanisms, such as neo-tracheogenesis or tumor growth, imply microenvironment modification with matrix modification. In mammals, this is especially induced by proteins called metalloproteases. These proteins are conserved in *Drosophila* with two MMP genes described, DmMmp1 and DmMmp2 [131,132]. These two MMPs possess distinct roles. MMP1 is implicated in trachea elongation and regulation of the circadian rhythm [133,134]. We also recently showed that MMP1 expression is associated with neo-tracheogenesis in *Drosophila* during accessory gland carcinogenesis induced by Ras^V12^ expression [128]. MMP2 is required for the ovulation process and regulation of WNT signaling [135,136]. MMPs also have common roles in the regulation of motoneurons growth, epidermal healing, coagulation, or basal lamina degradation during metamorphosis [137,138,139]. Because there are two MMPs in *Drosophila*, compared to the roughly 20 described in mammals, there is less redundancy, and it is easier to analyze their functions in this model by deletion, for example. Moreover, the presence of proteins able to induce modification of the microenvironment and cell adhesion suggests it is used for a more advanced tumorigenic phenomenon [140].

*Drosophila* can also be used to study the invasive capacities of tumor cells: Tumor transplantation can be done, where a primary tumor from a donor *Drosophila* is dissected and transplanted to an adult female abdomen. A few days later, the female host is dissected and the presence of tumor cells outside the abdomen in the thorax, head, legs, wings, muscle, brain, intestine, and ovaries will be considered as proof of cell migration capacities [104,119]. Moreover, if tumoral cells are found in ovarioles, this will be a proof of invasive capacities, as cells need to pass through two successive basement membranes and to reactivate MMP expression in order to do so [141].

#### 3.3.3. Use of *Drosophila* for Organotypic Models

Finally, *Drosophila* can be used for specific pathology and as organotypic *Drosophila* cancer models. We now know the importance of the cancer microenvironment and its interactions with the tumor. Some mutations or signaling pathway deregulations can drive cancer development in some tissues but have a very low impact in other ones [142]. This is what makes studies in a specific tissue so important. Of course, the same tissues in different species can also display different sensitivity to specific modifications, but nonetheless, in *Drosophila*, organotypic models have been established for glioma, colon, and lung cancer [129,143,144]. 

The gut has been widely used in *Drosophila* to model pathologies because it is considered as well conserved, with a similar function as mammals’ intestine (food digestion, nutrient absorption, and defense response against infection), as well as a similar structure [145]. In an article from 2016, *Drosophila* gut allowed for the production of multigenic models using data from The Cancer Genome Atlas, reproducing key features of human colon cancer and allowing drug screens to identify combinatorial therapy on specific genetic modifications [144]. The midgut also includes stem cells that share many characteristics with human intestinal stem cells, and have been used to identify new homeostasis control mechanisms, implicating stem cells that could be relevant in colorectal cancer [146,147].

Brain cancer is also widely studied in *Drosophila*. Several models have been made displaying different gene alterations: suppression of Brat expression to model glioma [148] and loss of function of lgl (lethal giant larvae) to model neurogenic brain tumors [149]. 

In addition to these studies on general processes of carcinogenesis, *Drosophila* can be used specifically for the study of prostate cancer thanks to the accessory glands, the functional equivalent of the prostate.

## 4. Accessory Glands as a Model of Epithelial Prostate Carcinogenesis

### 4.1. The Drosophila Accessory Glands, Functional Equivalents of the Prostate

The *Drosophila* reproductive tract is composed of structures with a similar function to that in men: two seminal vesicles, two testis, one ejaculatory tract, and two accessory glands [150]. Epithelial cells from the ejaculatory duct, seminal vesicles, and accessory glands secrete and allow for seminal fluid production. Accessory glands are the functional equivalent of the prostate. The main role of these glands is the secretion of proteins constituting the seminal fluid, such as proteases or glycoproteins [151,152,153,154], cysteine-rich proteins [155], and lectins [156,157]. As in men, secreted proteins can modulate bacteria resistance and immunity, particularly in the female genital tract [158,159]. In *Drosophila*, proteins such as Sex-peptide (SP or Acp70A, accessory gland protein 70A), Ovulin (Acp26Aa) [160], or CG33943 [161] have additional functions, such as increasing the female egg-laying rate and decreasing the attractivity of mated females for other males for a few days. 

In spite of these similar functions, important differences exist between *Drosophila* and human accessory glands. In *Drosophila*, accessory gland epithelial secretion depends on ecdysone [162], whereas in humans, it depends on testosterone, both of which are steroid hormones. However, ecdysone controls many more processes other than epithelial secretion, and is known as the molting hormone for its major role during the pupal stage of development [163,164]. Furthermore, the ecdysone receptor is more homologous to the liver X receptors than to the androgen receptor [165]. In this regard, it seems difficult to study in *Drosophila* the prostate cancer mechanisms that are directly dependent on androgen receptor signaling. Accessory glands roughly display a similar structure to a human prostate acinus. Each gland is composed of a monolayered epithelium made up of about 1000 cells surrounding a lumen, as compared to multilayered epithelium in humans. The *Drosophila* epithelium is composed of two types of cells: main or primary cells, which are flat and hexagonal, representing 96% of the epithelial secreting cells, and rarer secondary cells (about 4% of the cells), which are spherical and situated mostly at the extremity of the gland [166,167]. These epithelial cells are binucleated, due to incomplete mitosis (without cytokinesis), about 50 h after pupal formation [168]. In humans, the epithelium is mainly composed of two types of epithelial cells: the secretory luminal cells and the less differentiated basal cells. Rare neuroendocrine cells and intermediate epithelial cells are also intercalated between the basal cells. The *Drosophila* epithelial monolayer is surrounded by a thin layer of mononuclear striated muscle cells [169], which is itself enclosed in a basement membrane common to the epithelium [128], and so represents a stroma-like structure enclosing the epithelial compartment. During mating, muscle contraction allows seminal fluid expulsion from the lumen of the accessory gland to the female genital tract [170]. In humans, the fibromuscular stroma also contains endothelial cells, fibroblasts, and immune cells, and both epithelial and stromal compartments also have stem cells to allow for maintenance of the tissues. Epithelial human prostate is composed of three different zones, the peripheral one being the source of most of the cancers. So, morphologically, accessory glands represent a largely simplified version of the prostate, with furthermore a lower cell diversity. This, as for other models, represents both an inconvenience and an advantage: it limits modelization of the complex prostate microenvironment, which is crucial for the evolution of the human pathology. However, it also renders more accessible the interpretation of experiments done with this model. In this regard, it also provides a simple, easy to use in vivo model to study general mechanisms of epithelial tumorigenesis, such as basal extrusion, which is still poorly described due to the scarcity of adequate models to reproduce it experimentally.

Overall, several studies have then shown parallels between *Drosophila* accessory glands and human prostate epithelium, and proven their relevant use to study human prostate pathologies, such as prostate cancer [171,172,173,174].

### 4.2. Secondary Cells to Model Tumor Migration and Progression

In the accessory gland, the secondary cells can migrate by apical delamination, and this ability has been used to do a tissue-specific genetic screen directly in the accessory gland to discover new regulators of human cancer progression that promote growth and migration of secondary cells [171]. After this first screen, the interesting genes were tested in human prostate cells to confirm their relevance. Moreover, abundant microvesicles are present in these secondary cells, and secreted as exosomes. The accessory gland has been demonstrated as a useful model to study mechanisms regulating these secretions, which should be of interest, considering the importance of exosomes in carcinogenesis [172]. Indeed, these microvesicles secreted in the prostate from the endosomal multivesicular body (MVB) can fuse with sperm to modulate its activity and reinforce its homeostasis [154]. They are implicated in multiple aspects of cancer biology because of their capacity to secrete metabolites and growth factors, ultimately aiding tumor growth. They are also responsible for increased drug resistance by activating mechanisms allowing the elimination of toxic chemicals, such as chemotherapeutic products [175,176]. Moreover, we know that during prostate carcinogenesis, there is a switch from a hormone-dependent to hormone-independent status of prostate cancer cells, and this can lead to CRPC. In *Drosophila*, this switch from a hormone-dependent to hormone-independent status exists in the secondary cells of accessory glands. A parallel has been made by this model to the hormone-independent status in human prostate cancer progression [173]. Thus, the use of secondary cells in the accessory gland opens many perspectives to decipher the molecular mechanisms implicated in prostate cancer.

### 4.3. The Drosophila Accessory Glands as a Model for Basal Epithelial Cell Extrusion

Most human cancers present an epithelial origin, such as prostate cancer [85]. A key step in tumorigenesis is the ability of epithelial cells to leave their compartment, allowing the formation of primary tumors, and preceding formal invasion leading to the formation of metastases at distant sites. For this, epithelial cells must cross the basement membrane, a phenomenon known as epithelial basal extrusion. Understanding the mechanisms involved in this key step could help prevent tumor progression and metastasis. As a reminder, the 5-year survival of patients with non-invasive prostate cancer is close to 100% and drops to 30% when prostate cancer has invaded other areas [177]. However, this step is elusive enough, and only rare articles have studied which signaling pathways could be involved in this phenomenon. This is why we recently developed a unique in vivo model of tumorigenesis in the *Drosophila* accessory glands, allowing the study of basal extrusion [128]. The clonal expression of an oncogene, Ras^v12^, mimicked initiation, and was able to induce a tumorigenic process recapitulating several key features of prostate cancer: cell hyperproliferation and hypertrophy, neo-tracheogenesis facilitating oxygen supply for the tumor cells, and loss of epithelial markers and thus loss of epithelial identity. The latter is a phenomenon notably observed during epithelial-mesenchymal transition (EMT), which is essential in tumorigenesis, and more importantly in basal extrusion. Indeed, the specific shape of the accessory gland allows for easy observation of tumors forming outside the epithelium, following basal extrusion of tumor cells. The use of a large number of animals even allows quantification of this phenomenon. Thus, this model has allowed for the more precise description of the role of two major signaling pathways in the initiation of prostate cancer: the RAS/MAPK and PI3K/AKT/TOR pathways. Although these pathways were well known to be deregulated and involved in prostate cancer progression, their involvement in the early phases of tumorigenesis remained poorly understood. We showed that RAS/MAPK and PI3K/AKT/TOR pathways cooperate to induce basal extrusion and thus tumor formation. Their coactivation involves the sequential recruitment of two feedback loops dependent on two growth factors: EGF (Spitz) and IGF (Ilp6), and their respective receptors. These results obtained in *Drosophila* led to public bioinformatics data analysis and in vitro tests on transformed human prostate cells, validating the possible involvement of the same pathways in early human prostate carcinogenesis.

Finally, due to the almost complete lack of knowledge on basal extrusion and the fact that several hallmarks of cancer are common independently of the origin of this pathology, this model could be of interest for other epithelial cancers.

## 5. Conclusions

Improving the management of prostate cancer patients requires a better understanding of the players and mechanisms involved at each stage of the disease. For this, studies on in vitro and in vivo models are necessary. Each model, whether cellular, murine, or *Drosophila*, brings different approaches and different perspectives, making them complementary. In a first approximation, some models seem better suited than others to study specific aspects of prostate cancer biology (see Table 1 and Table 2). While cellular models are an excellent first approach for the study of biological processes in which we can easily study molecular interactions, they are still mostly based on 2-D models in which we cannot reproduce human pathology. The 3-D cell models allow better reproduction of the tumor microenvironment by combining the presence of several cell lines and a three-dimensional structure, reproducing essential cellular interactions in human pathology. Moreover, they can be developed directly from patient biopsies, allowing pre-clinical studies to be performed. However, the need to use aggressive cells to obtain them limits their use to the study of late stages of tumorigenesis. Murine models have the advantage of reproducing human pathology with its different stages as well as the interactions between cancer cells, the stroma, and the microenvironment. They are therefore very useful in the study of genes or groups of genes in the tumorigenic process, from initiation to the later stages of tumorigenesis. However, in genetic models of overexpression/deletion, initiation is only imperfectly reproduced as genetic modification occurs in a large proportion of the epithelial cells. When it comes to xenograft models, the tumor microenvironment is not necessarily adequate, especially with an altered immune system. In any case, in mice, the development of tumors can take time, cost a lot of money, and some can argue that a limited number of successful clinical trials have validated this model so far. With the emergence of 3Rs regulation, pressure to decrease the use of such models has increased despite the interest in cancer research. For this, the use of *Drosophila* provides a new perspective to possibly better understand the mechanisms involved in prostate cancer. Its main strength relies on the fact that it stays an in vivo model in which fundamental cellular processes and signaling pathways are well conserved. It allows for rapid, simple studies thanks to the numerous genetic tools available, and at a lower cost. It also allows for the study of tumorigenic stages that are difficult to study in other models, as it is the case for basal extrusion.

To conclude, we showed here that the *Drosophila* accessory gland represents a potent new model to modelize prostate tumorigenesis as well as study specific steps of general epithelial tumorigenesis, such as basal extrusion in vivo. It is difficult to summarize, as we tentatively propose in Table 1 and Table 2, both the many events associated with tumorigenesis, and the richness of opportunities brought by biological models. We suggest that *Drosophila* will illustrate how new knowledge can be gained in unexpected ways. As said previously, despite having no equivalent of the androgen receptor in this insect, new partners of this receptor were found using the S2 cell line [110]. In the end, the review of the literature indicates that the important thing is to have available the largest panel of models, in the hope of understanding cancer biology in its vast diversity.

## Figures and Tables

**Table 1:** Use of different prostate cancer models.

		Prostate Normal Growth and Development	Non Tumorigenic Prostatic Pathologies	Early Prostate Carcinogenesis	Androgen-Insensitive Transition	Late Prostate Cancer (CRPC) and Metastasis	Pre- Clinical Studies
**2-D cell lines**							
Untransformed	RWPE-1, BPH-1, PRNS-1-1, P69						
Androgen-sensitive	LNCaP, LAPC-4, LAPC-9, LuCaP 23.1						
Androgen-insensitive	PC3, DU145						
**3-D models**							
**Mouse** **models**	Xenograft						
	Genetic models						
***Drosophila* models**							

The choice of an appropriate study model is crucial to answer a biological question in a relevant way. Each type of model is represented here with a color code from green to red in order to have an overview of the existing models and their optimal use. Green corresponds to a stage that can be studied in the corresponding model, contrary to red, where the model is not adequate. Orange represents intermediate adequacy of a given model for a given question.

**Table 2:** Main advantages and disadvantages of the considered models.

	Advantages	Disadvantages
2-D Cell lines	- Provide functional and mechanistic insight - Well characterized - Easy to obtain and manipulate	- Monolayer culture - Inability to reproduce the pathology - Majority of cell lines derived from metastases: limit their use for early prostate cancer - Absence of tumor microenvironment
3-D models	- Closer to a native tumor - Better conservation of heterogeneity - Partial tumor microenvironment - Multilayer culture - Conservation of cell morphology - Conservation of cell-cell and cell-matrix interactions	- Still in development, not a routine procedure - Remain ex vivo (metastatic studies limitation, limited microenvironment) - Use of aggressive cells: not adapted for early prostate cancer studies
Mouse Xenografts models	- Tumor easily accessible - Can be done with benign and malignant tissue (SRC) - Transplantation in a definite organ, metastatic potential can be evaluated (SRC) - Conserved interactions between implanted tissue and prostate microenvironment (ortho) - Can generate metastases - In vivo - Conservation of prostate tumor heterogeneity (PDX models)	- Can take several months to develop a tumor - Non prostatic microenvironment for subcutaneous and SRC transplantation - Only high-grade tissue transplantation for subcutaneous xenograft - Used of immunodeficient mice - Loss of prostate tumor heterogeneity
Mouse genetic models	- Intact prostate microenvironment - Temporally observation of gene manipulation and drug treatment - Preservation of most of histopathological features observed in human pathology - In vivo	- Use of androgen-dependent promoter - Differential organization of mouse prostate compared to human - Tumor development can take several months - Limitation of the number of animals that can be used (3Rs). - Gene redundancy complicating signaling pathways studies - Low success of clinical trials emerging from mice
*Drosophila* models	- Short life cycle (10 days at 25 °C) - Large number of offspring per generation - Well-described anatomy - Huge amounts of genetic tools available - Few redundancies and good conservation of fundamental biological mechanisms and signaling pathways - Acinus-like organization	- Different microenvironment - Far from mammals: results ought to be confirmed in cell models. - No ortholog of the androgen receptor
